# Optimization of Coconut Fiber in Coconut Shell Concrete and Its Mechanical and Bond Properties

**DOI:** 10.3390/ma11091726

**Published:** 2018-09-14

**Authors:** Anandh Sekar, Gunasekaran Kandasamy

**Affiliations:** Department of Civil Engineering, Faculty of Engineering and Technology, SRM Institute of Science and Technology, Chennai 603203, India; gunasekaran.k@ktr.srmuniv.ac.in

**Keywords:** coconut shell, aggregate, coconut fiber, mechanical properties, bond properties

## Abstract

Coconut shell concrete is one of the recently established lightweight concretes. This paper discusses the optimization of adding coconut fibers in both coconut shell concrete and conventional concrete. Coconut fibers at different aspect ratios of 16.67, 33.33, 50, 66.67, 83.33, and 100 and volume fractions of 1, 2, 3, 4, and 5% were tried. The maximum compressive strength was attained at an aspect ratio of 83.33 and volume fraction of 3% for conventional concrete, and aspect ratio 66.67 and volume fraction 3% for coconut shell concrete. Flexural strength increased by 30.63% (conventional concrete) and 53.66% (coconut shell concrete) on the addition of coconut fibers. Similarly, the split tensile strength increased by 19.44% and 30%, respectively. The number of blows needed for failure of specimen in impact resistance test was more for concrete mixed with fibers. The experimental bond stresses were higher than the theoretical values recommended by IS 456: 2000 (Indian Standard) and BS 8110 (British Standard). This study shows that the addition of coconut fiber enhances the properties of both conventional and coconut shell concrete.

## 1. Introduction

In general, concrete is strong in compression and weak in tension. Plain concrete possesses a very low tensile strength, limited ductility, and little resistance to cracking. Internal microcracks are inherently present in the concrete and its poor tensile strength is due to propagation of such microcracks, eventually leading to the brittle failure of the concrete. Conventionally the tensile properties of concrete members are improved by the reinforcement with steel. However this does not increase the inherent tensile strength of concrete itself. In plain concrete, microcracks develop even before loading. After loading, the microcracks propagate, and stress concentration results in additional cracks where there are minor defects in the concrete. The development of such microcracks is the main cause of inelastic deformations in concrete. It has been recognized that the addition of small, closely spaced, and uniformly dispersed fibers to concrete would act as crack arresters [1]. Recent works on the use of different fibers in concrete indicates that the fibers enhance the properties of concrete [2,3,4,5,6,7]. Type of fibers used in concrete matrix influences the structural responses and also in design [8,9]. Coconut shell has been recently established as viable material in the production of concrete and its properties as an aggregate and in structural and non structural members have been published [10,11,12,13,14,15,16,17,18]. This paper explores the effects of adding coconut fiber to coconut shell concrete. The mechanical properties such as compressive, flexural, splitting tensile strengths, and impact resistance after the addition of coconut fiber are analyzed and results are reported. Since bonding between the concrete and the reinforcements is also equally important for any new combination of concrete, bond tests were also conducted. Coconut fibers were also added to conventional concrete (CC) and the properties were studied for comparison.

## 2. Materials Used

**Figure 1:**
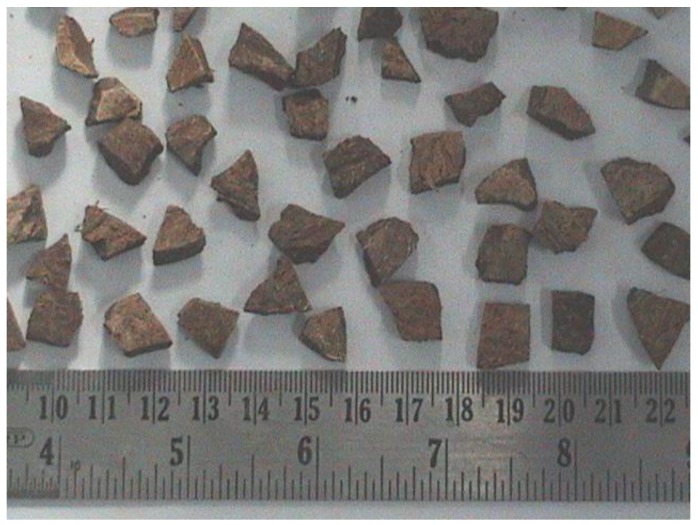
Coconut shell aggregate.

Ordinary Portland cement (OPC) conforming to the Indian Standard IS 12269: 1987 [19] was used throughout this study. River sand of maximum size 4.75 mm, obtained from the local river Palar and conforming to grading zone III as specified in IS 383: 1970 [20] was used as fine aggregate. Crushed coconut shells (Figure 1) and conventional stones of a maximum size of 12.5 mm were used as coarse aggregates. Table 1 illustrates the physical properties of aggregates used in this study.

**Figure 2:**
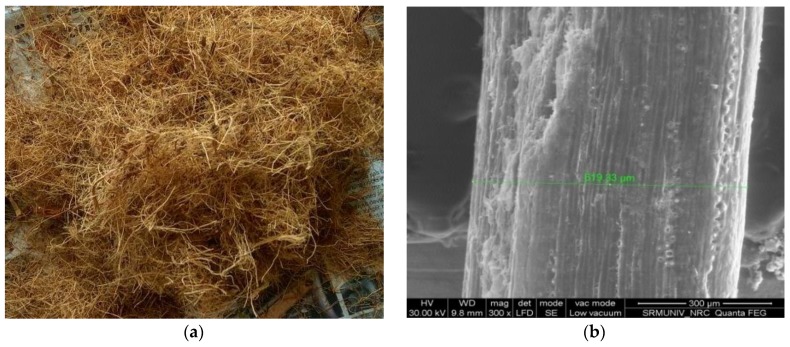
(**a**) Coconut fibers used in this study; and (**b**) Typical SEM Image of coconut fiber.

Coconut fibers used were procured from the local coconut industries (Shanmugam Traders, Kancheepuram, India) (Figure 2a). Different aspect ratios (16.67, 33.33, 50, 66.67, 83.33, and 100) and volume fractions (1%, 2%, 3%, 4%, 5%, and 6%) of coconut fibers were tried in order to achieve the maximum compressive strength. The optimum aspect ratio and volume fraction that gave the maximum compressive strength was used for further studies. Scanning electron microscope (SEM) images showed that the average diameter of coconut fiber was 0.6 mm. A typical SEM image is shown in Figure 2b.

## 3. Experimental Procedure

For both CC and coconut shell concrete (CSC), a compressive strength of 25 N/mm^2^ at 28 days was targeted. Mix ratio used for CC was 1:2.22:3.66:0.55 with a cement content of 320 kg/m^3^, and for CSC, it was 1:1.47:0.65:0.42 with a cement content of 510 kg/m^3^ [11,12]. These mix proportions were used as controlled mixes without the addition of coconut fibers. Then, coconut fibers were added at different aspect ratios of 16.67, 33.33, 50, 66.67, 83.33, and 100 and volume fractions of 1%, 2%, 3%, 4%, 5%, and 6% to find the optimum mix that produced the maximum compressive strength. Cubes of size of 100 × 100 × 100 mm were tested according to IS 516: 1959 [21] to determine the compressive strength of concrete.

To determine the flexural resistance, the standard size of the specimen used was 100 × 100 × 500 mm. This test was conducted by the application of four points loading as per ASTM C78-84 (American Society for Testing and Materials) [22]. The flexural strength was calculated as PLbd2, where P is maximum load applied in N, L is supported length of specimen in mm, b is the width of specimen in mm, and d is the depth of specimen in mm.

To determine the split tensile strength, the standard size of the specimen used was 100 mm in diameter and 200 mm height. This test was conducted as per ASTM C496-90 [23]. The split tensile strength was calculated as 2PπDL, where P is maximum load applied in N, D is cross sectional diameter of the specimen in mm, and L is the length of specimen in mm.

To determine the impact resistance, the size of the specimen used was 152.4 mm in diameter and 63.5 mm thick. This test was conducted as per the method developed by ACI committee 544.1R-82 (American Concrete Institute). During the impact test, the number of blows was counted till the first crack appeared (initial crack) on each specimen and counting was continued till the specimen was broken (final crack) into a number of pieces. Though the tests were conducted at the age of three days, seven days, and 28 days, only the 28 days result is presented.

**Figure 3:**
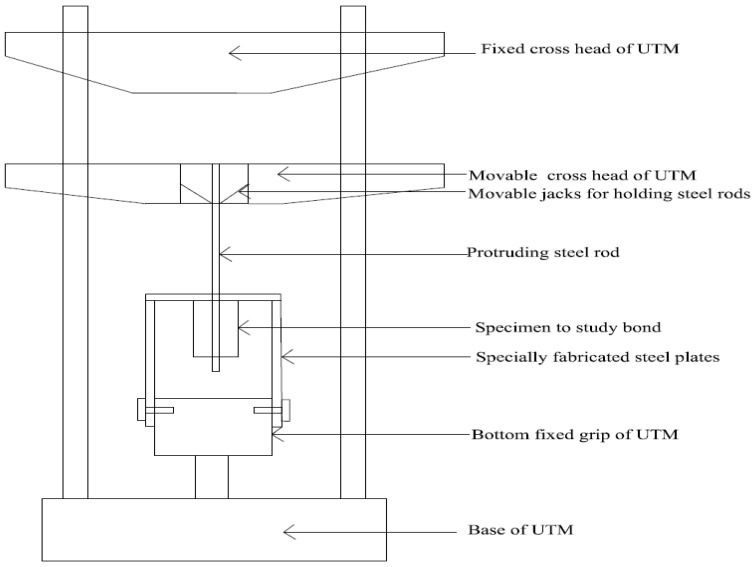
An arrangement for testing bond specimen in UTM.

To study the bond stress, a pull out test was conducted as per IS 2770 (Part-I 1987) [24], and the bond strength was calculated. The size of the specimen used was 100 mm in diameter and 200 mm height. Both plain and deformed bars having diameters of 8, 10, 12, and 16 mm were used in CC and CSC mixes and tested for bond strength with and without addition of coconut fibers. The bond stress was calculated as ι = Fπdl where ι is the bond stress in N/mm^2^, *F* is the applied load in N, *d* is the nominal bar diameter in mm, and *l* is the effective bonded length (150 mm). The bond stress reported is the average of three specimens in each case. The line diagram of the universal testing machine (UTM, Fyne Spavy Associates, Miraj, India) modified for testing the bond strength is shown in Figure 3.

Table 2 illustrates the different test parameters considered, number of specimen used for each parameter, and the age of specimen during testing.

## 4. Results and Discussion

### 4.1. Properties of Fresh and Hardened Concrete Without Fibers

The slump was 10 mm for both CC and CSC mixes without coconut fibers. The density of fresh CC and CSC mixes were found as 2520 kg/m^3^ and 1957 kg/m^3^, respectively. Densities of hardened concrete of CC and CSC were 2510 kg/m^3^ and 1952 kg/m^3^ at three days; 2515 kg/m^3^ and 1955 kg/m^3^ at seven days; and 2527 kg/m^3^ and 1968 kg/m^3^ at 28 days, respectively. Compressive strengths of CC and CSC mixes were 19.6 N/mm^2^ and 18.3 N/mm^2^ at three days; 21.9 N/mm^2^, and 20.4 N/mm^2^ at seven days; and 30.1 N/mm^2^ and 25.6 N/mm^2^ at 28 days, respectively.

### 4.2. Properties of Fresh and Hardened CC and CSC with Fibers

**Figure 4:**
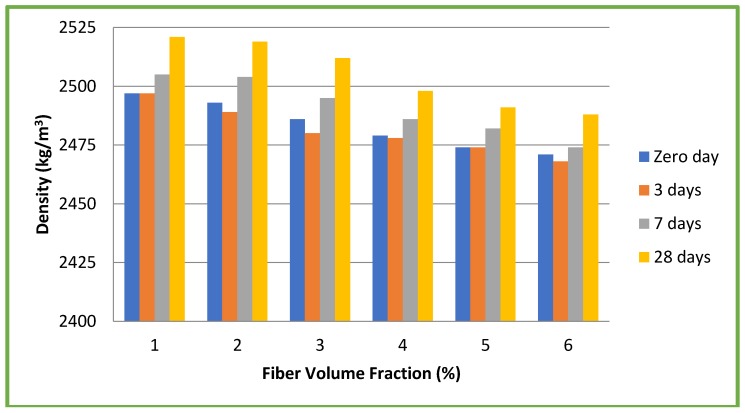
Densities of conventional concrete mixes.

**Figure 5:**
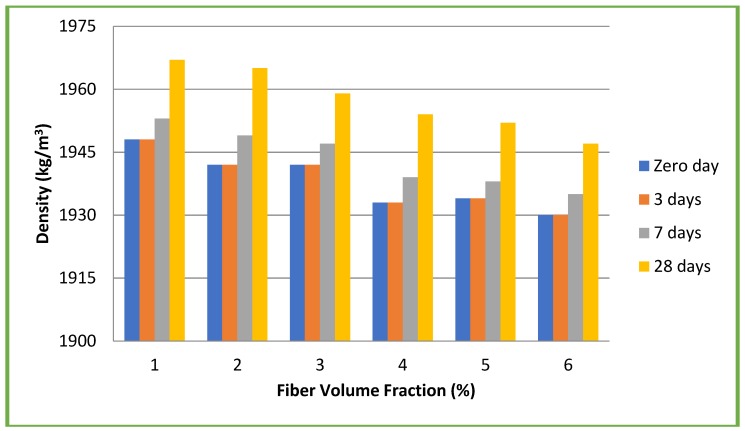
Densities of coconut shell concrete mixes.

Since fibers were added on volume basis, aspect ratio did not influence the densities of the mixes used. Therefore, the slump values were eight–10 mm for CC and six–10 mm for CSC mixes and no difficulties were faced during casting of specimens and compaction. Both the slump and density decreased with increase in the percentage of fibers. The fibers play a role in influencing the workability and density of both CC and CSC. Fresh (zero day) and hardened concrete densities of both CC and CSC mixes at three, seven and 28 days for different volume fractions (1, 2, 3, 4, 5, and 6%) irrespective of the aspect ratios are given in Figure 4 and Figure 5.

### 4.3. Optimization of Coconut Fibers in CC and CSC Mixes

**Figure 6:**
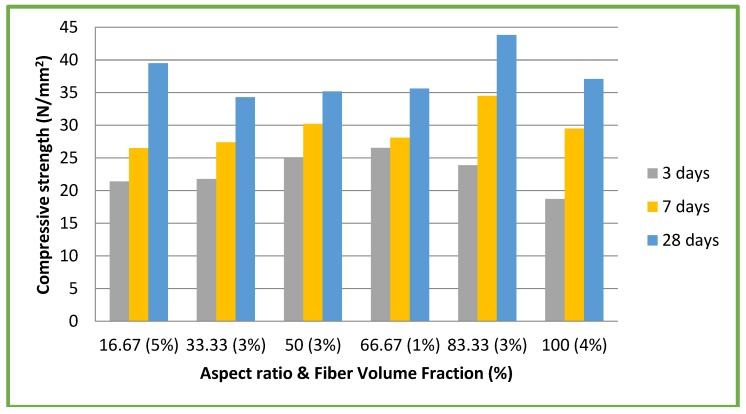
CC mixes: compressive strength vs aspect ratio & volume fraction (%).

Thirty one mixes for CC and 25 for CSC with different aspect ratios and volume fractions were tested for compressive strength. For each mix, nine cubes were cast, of which, three cubes each were tested at three, seven, and 28 days and the average was reported. Table 3 shows the results for CC mixes. The maximum compressive strength corresponding to a particular aspect ratio and volume fraction is highlighted in bold. Figure 6 shows the results of compressive strength test for all CC mixes. The maximum compressive strength of 43.8 N/mm^2^ was achieved for aspect ratio of 83.33 and volume fraction of 3% (Figure 6). This value is 45.51% more, as compared to the compressive strength of CC mix without coconut fibers (30.1 N/mm^2^).

**Figure 7:**
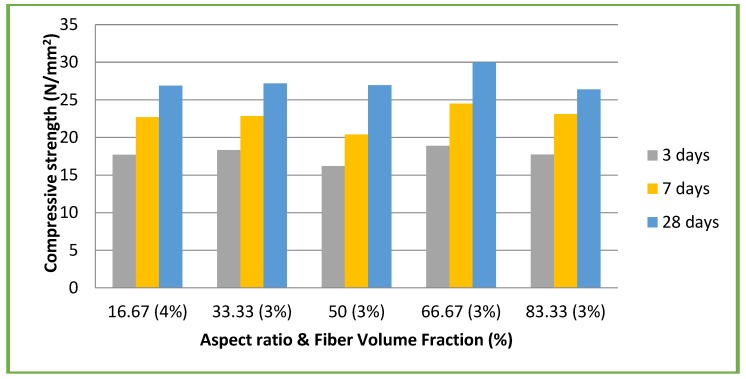
CSC mixes: compressive strength vs aspect ratio & volume fraction (%).

Similarly the results of CSC mixes with different aspect ratios and volume fractions are given in Table 4. Figure 7 shows the results of compressive strength test for all CSC mixes. The maximum compressive strength of 30.01 N/mm^2^ was achieved for an aspect ratio of 66.67 and volume fraction of 3% (Figure 7). This value is 17.22% more compared to the compressive strength of CSC mix without coconut fibers (25.6 N/mm^2^).

### 4.4. Flexural Strength

**Figure 8:**
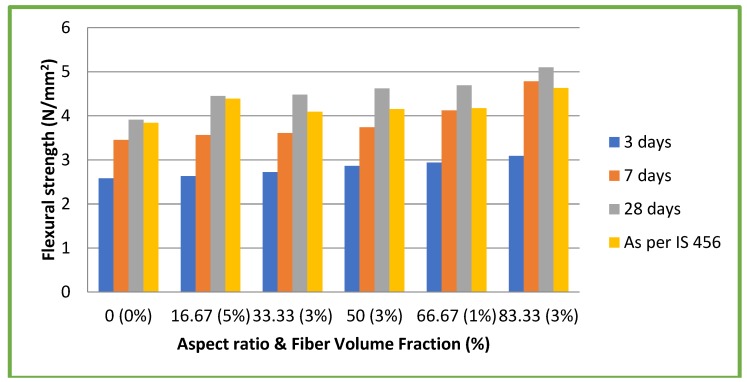
Flexural strength test results of CC mixes.

**Figure 9:**
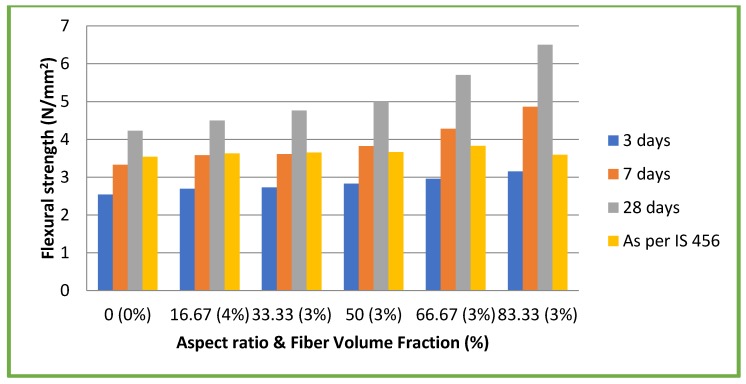
Flexural strength test results of CSC mixes.

Figure 8 and Figure 9 illustrate the results of flexural strength tests on both CC and CSC mixes, respectively. In the case of CC mixes, maximum flexural strength of 5.10 N/mm^2^ (11.64% of compressive strength) was attained at 28 days for the aspect ratio 83.33 with a 3% volume fraction. This is an increase of 30.43% compared to CC without fiber (3.91 N/mm^2^). For CSC mixes, maximum flexural strength of 6.50 N/mm^2^ (24.62% of compressive strength) was attained at 28 days for the aspect ratio 83.33 with 3% volume fraction. This is an increase of 53.66% to CSC without fiber (4.23 N/mm^2^). The experimental results at 28 days were compared with the theoretical formula recommended by IS 456: 2000 [25] 0.7 $\surd f_{ck}$, where $f_{ck}$ = compressive strength of concrete. In all the cases, experimental values were found to be higher than the theoretical values. Though the flexural strength of concrete is related to its compressive strength, the addition of coconut fibers (aspect ratio) had a different effect on the flexural strength of both CC and CSC mixes (Figure 8 and Figure 9). The flexural strength increase with increase in aspect ratio, whereas the compressive strength did not show such a clear relation. Additionally, though the compressive strengths of CSC mixes were lower compared to CC mixes, the CSC exhibited higher flexural strength. This is due to fibrous nature of the coconut shell. The same trend was observed in the earlier studies on coconut shell concrete [10,26].

### 4.5. Split Tensile Strength

**Figure 10:**
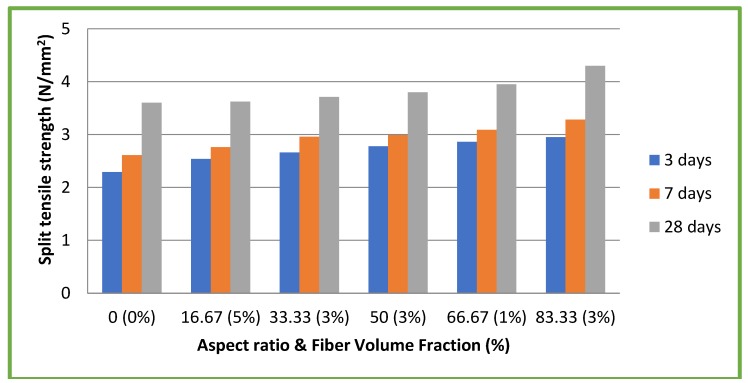
Split tensile strength test results of CC mixes.

**Figure 11:**
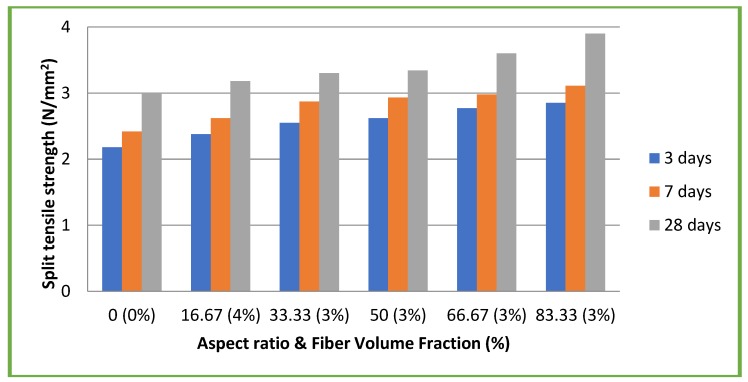
Split tensile strength test results of CSC mixes.

Figure 10 and Figure 11 illustrate the results of split tensile strength tests on both CC and CSC mixes, respectively. In case of CC mixes, maximum split tensile strength of 4.30 N/mm^2^ (9.81% of compressive strength) was attained at 28 days for the aspect ratio 83.33 with a 3% volume fraction. This is an increase of 19.44% compared to CC without fiber (3.6 N/mm^2^). For CSC mixes, maximum split tensile strength of 3.90 N/mm^2^ (14.77% of compressive strength) was attained at 28 days for the aspect ratio 83.33 with 3% volume fraction. This is an increase of 30% compared to CSC without fiber (3 N/mm^2^). This shows that the use of coconut fiber in CC and CSC mixes augments the split tensile strength of the concrete. These results also emphasize the significant role of the aspect ratio.

### 4.6. Impact Resistance

**Figure 12:**
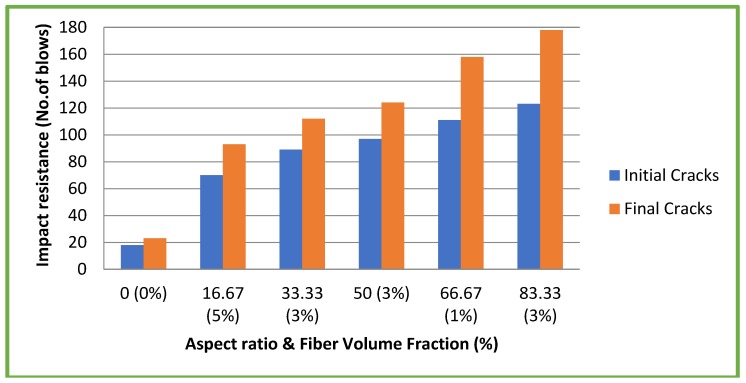
Impact resistance test results of CC mixes.

**Figure 13:**
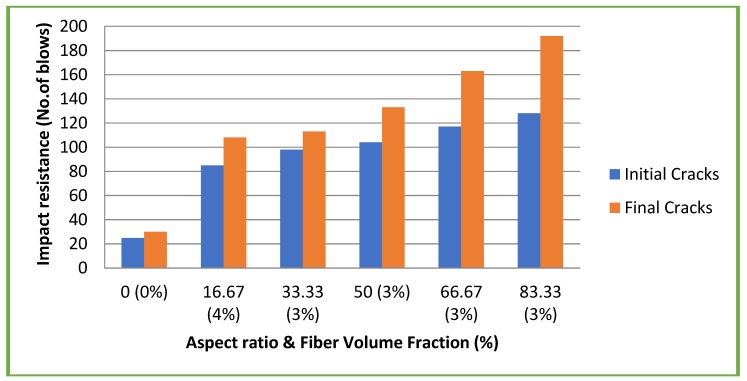
Impact resistance test results of CSC mixes.

Figure 12 and Figure 13 illustrate the results of impact resistance tests at 28 days on both CC and CSC mixes, respectively. In general, the impact resistance increases with concrete strength. There is an optimum value of impact resistance in normal concrete beyond which any increase in strength does not play any role in impact resistance both at first crack and at final crack [27]. Literature also shows that the failure of conventional concrete having compressive strength of around 45 N/mm^2^ requires only 10–22 blows [27]. This is reinforced in this study as the CC mix without fibers have compressive strength 30.1 N/mm^2^ that failed at 18–23 blows. As the aspect ratio increased, the number of blows required for initial cracks and final failures of CC with fibers that also increased. The CSC mixes also behaved in a similar manner. However, in general, the impact resistance of CSC mixes both with and without fibers is higher compared to CC mixes with and without fibers. This increase in impact resistance of CSC mixes may be due to the fibrous nature of the CS aggregate and its high impact resistance.

### 4.7. Bond Properties

**Figure 14:**
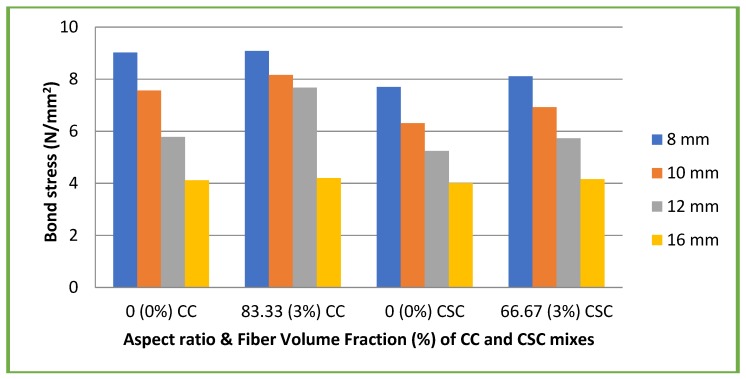
Bond stress of plain bars with different mixes.

**Figure 15:**
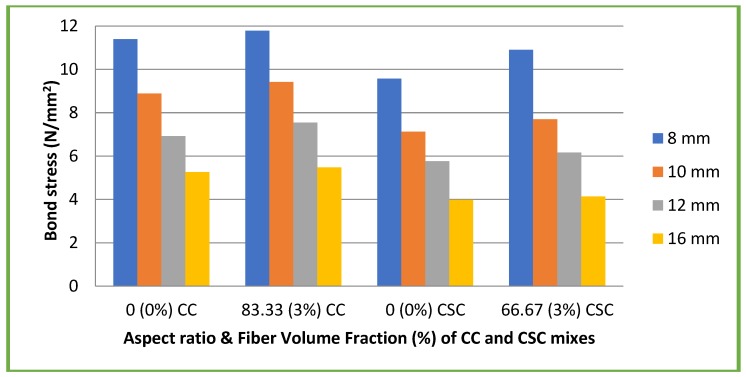
Bond stress of deformed bars with different mixes.

Figure 14 and Figure 15 illustrate the results of bond stress at 28 days of both plain and deformed bars with different mixes.

Theoretical bond strengths were calculated as per IS 456: 2000 [25] and BS 8110 [28] (Table 5).

In case of plain bars, bars come out of the specimen without forming any visible cracks over the surface. However, in the case of deformed bars, specimens fail suddenly after the formation of longitudinal cracks. The outcrop on the exterior of the deformed bars plays a significant role in improving the bond strength.

In case of CC mixes, the bond strength of specimens with plain bars ranged from 4.12 to 9.02 N/mm² (13.68 to 29.9% of its compressive strength) and in the case of CSC mix, the bond strength of specimens with plain bars ranged from 4.01 to 7.7 N/mm² (15.6 to 30% of its compressive strength). Similarly for CC mixes, the bond strength of specimens with deformed bars ranged from 5.26 to 11.39 N/mm² (17.4 to 37.8% of its compressive strength) and for CSC mixes, the bond strength of specimens with deformed bars ranged from 3.98 to 9.57 N/mm² (15.54 to 37.38% of its compressive strength).

In the case of CC mixes with coconut fiber, the bond strength of specimens with plain bars ranged from 4.2 to 9.08 N/mm² (9.5 to 20% of its compressive strength) and in the case of CSC mixes with coconut fiber, the bond strength of specimens with plain bars ranged from 4.16 to 8.11 N/mm² (13.8 to 27.02% of its compressive strength). Similarly for CC mixes with fiber, the bond strength of specimens with deformed bars ranged from 5.47 to 11.78 N/mm² (12.48 to 26.8% of its compressive strength) and for CSC mixes with fiber, the bond strength of specimens with deformed bars ranged from 4.14 to 10.9 N/mm² (13.79 to 33.6% of its compressive strength).

In general, the addition of coconut fibre enhances the bond strength. The experimental results were more than the theoretical bond strength recommended by both IS 456: 2000 [25] and BS 8110 [28]. The bond strength decreased as the diameter of the bar increased, whether a plain bar or deformed bar, which is similar to earlier studies [10,26]. This is because of the volume of concrete and confining pressure on the steel bars.

### 4.8. Post-Cracking Behaviour

Ideally, CC specimens without fibers fail by crushing during compressive test and the cubes do not change shape. But the CSC specimens without fibers bulged slightly after the formation of initial cracks. The addition of coconut fibers delayed the propagation of failure of both CC and CSC specimens. The CSC specimens bulged slowly after the formation of initial cracks and the propagation of final failure was delayed. Hence, it can be concluded that the addition of coconut fibers can enhance the ductility properties of concrete, which is an advantage when it is used for earthquake resistant structures.

**Figure 16:**
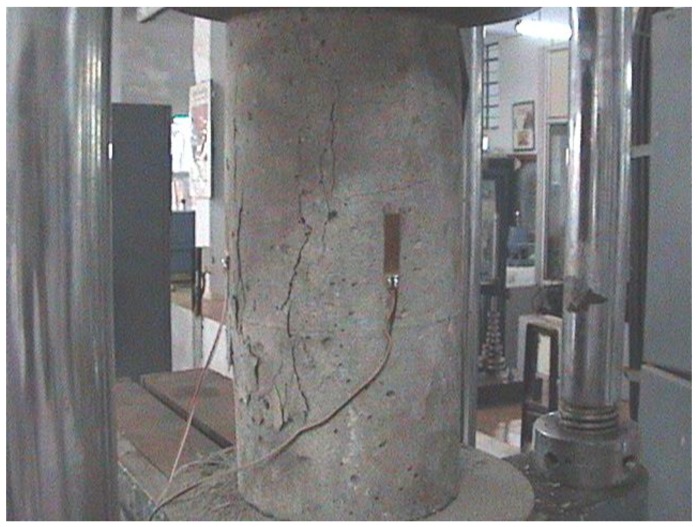
Bulged shape of CSC cylinder during test.

The bulging nature of the mix was additionally examined on a cylinder specimen (CSC mix with coconut fibers). Figure 16 shows the bulging of the cylinder specimen. More studies are needed on CSC structural elements with coconut fiber under both static and dynamic loading to confirm the effect of coconut fibers on the ductility of concrete.

## 5. Conclusions

Both conventional concrete and coconut shell concrete mixes were studied with and without the addition of coconut fiber. In both CC and CSC mixes, as the percentage of fibers increases, workability and density decrease. From the compressive strength test results, it can be stated that the coconut fiber, having an aspect ratio of 83.33 and a volume fraction of 3%, is the optimum that can be used in CC mix to achieve the maximum strength for the mix proportion used in this study. Similarly, coconut fiber having an aspect ratio 66.67 and a volume fraction of 3% is the optimum that can be used in CSC mix to achieve the maximum strength for the mix proportion used in this study.

Maximum flexural strengths of CC and CSC mixes added with coconut fibers were 30.63% and 53.66% more than that of CC and CSC mixes without coconut fibers, respectively. The aspect ratio of coconut fibers plays a significant role in the improvement of flexural strength. Additionally, flexural strength increased with the aspect ratio.

Maximum split tensile strengths of CC and CSC mixes added with coconut fibers were 19.44% and 30% more than that of CC and CSC mixes without coconut fibers, respectively. Split tensile strength test results also show that the aspect ratio plays a significant role.

The impact resistance of CSC mixes both with and without fibers is higher compared to CC mixes. The addition of coconut fiber to both CC and CSC enhances the bond strength. The experimental bond strengths were more than the theoretical values recommended by both IS 456: 2000 and BS 8110 standards.

This study proves that addition of coconut fibers enhances the properties of CC and CSC. However, further studies are required on other properties like durability, temperature resistance, and structural behaviour under different types of loading.

## Figures and Tables

**Table 1:** Physical properties of aggregates.

Physical and Mechanical Properties	Crushed Stone Aggregate	Coconut Shell	River Sand
Maximum size (mm)	12.50	12.50	4.75
Water absorption (%)	0.25	23.50	-
Specific gravity	2.67	1.15	2.61
Fineness Modulus	6.76	6.55	3.72
Bulk density (kg/m^3^)	1620	655	1695
Crushing value (%)	18.44	2.62	-
Impact value (%)	15.70	4.72	-

**Table 2:** Experiments and number of samples used in this study.

Sl. No	Test Parameter	Number of Specimens	Age during Testing
1	For compressive strength test—optimization	For CC—31 mixes; 9 cubes in each mix; total of 279 cubes. For CSC—25 mixes; 9 cubes in each mix; total of 225 cubes.	3 days, 7 days and 28 days 3 days, 7 days and 28 days
2	Flexural strength test	For CC—6 mixes; 9 beams in each mix; total of 54 beams. For CSC—6 mixes; 9 beams in each mix; total of 54 beams.	3 days, 7 days and 28 days 3 days, 7 days and 28 days
3	Split tensile test	For CC—6 mixes; 9 cylinders in each mix; total of 54 cylinders. For CSC—6 mixes; 9 cylinders in each mix; total of 54 cylinders.	3 days, 7 days and 28 days 3 days, 7 days and 28 days
4	Impact strength test	For CC—6 mixes; 9 specimens in each mix; total of 54 specimens. For CSC—6 mixes; 9 specimens in each mix; total of 54 specimens.	3 days, 7 days and 28 days 3 days, 7 days and 28 days
5	Bond strength test	For CC—4 mixes; 48 specimens in total. For CSC—4 mixes; 48 specimens in total.	28 days 28 days

**Table 3:** Results of CC mix with different aspect ratios and volume fractions.

Aspect Ratio	% of Fiber	Compressive Strength, N/mm^2^		
		3 Day Strength	7 Day Strength	28 Day Strength
16.67	1	16.80	22.20	31.40
	2	17.30	23.00	32.70
	3	18.90	24.80	34.60
	4	21.00	26.30	37.00
	**5**	**21.40**	**26.50**	**39.50**
	6	20.90	26.00	37.50
33.33	1	19.86	25.70	32.50
	2	20.53	26.66	33.73
	**3**	**21.76**	**27.40**	**34.30**
	4	18.23	21.90	29.20
	5	17.60	20.70	25.80
50	1	20.61	22.89	29.90
	2	23.45	27.33	32.13
	3	25.12	30.20	35.17
	4	21.01	27.78	33.98
	5	16.30	25.81	29.52
66.67	**1**	**26.53**	**28.10**	**35.63**
	2	21.57	24.97	33.46
	3	19.33	23.37	33.16
	4	16.71	19.23	32.01
	5	14.60	16.63	27.93
83.33	1	17.87	23.70	30.10
	2	21.30	27.03	36.97
	**3**	**23.90**	**34.50**	**43.80**
	4	15.36	23.03	32.03
	5	14.90	21.90	28.41
100	1	16.90	23.10	31.20
	2	17.40	26.10	33.10
	3	17.63	27.90	34.90
	**4**	**18.70**	**29.50**	**37.10**
	5	15.61	24.00	32.50

**Table 4:** Results of CSC mix with different aspect ratios and volume fractions.

Aspect Ratio	% of Fiber	Compressive Strength, N/mm^2^		
		3 Day Strength	7 Day Strength	28 Day Strength
16.67	1	16.20	20.00	26.00
	2	16.30	21.30	26.20
	3	16.50	21.90	26.50
	**4**	**17.70**	**22.70**	**26.90**
	5	15.90	20.70	24.40
33.33	1	16.80	18.10	21.30
	2	17.00	19.76	23.60
	**3**	**18.33**	**22.84**	**27.20**
	4	17.76	21.83	25.30
	5	17.66	19.13	23.50
50	1	13.70	18.60	26.00
	2	15.60	19.20	26.30
	**3**	**16.20**	**20.40**	**26.95**
	4	12.90	18.40	25.91
	5	11.40	18.30	24.00
66.67	1	15.20	19.90	25.87
	2	16.80	21.89	27.80
	**3**	**18.90**	**24.49**	**30.01**
	4	16.20	22.14	26.78
	5	15.90	21.09	25.10
83.33	1	17.00	22.90	25.20
	2	17.10	22.98	26.00
	**3**	**17.73**	**23.11**	**26.40**
	4	16.61	21.19	24.90
	5	16.31	20.03	24.85

**Table 5:** Theoretical bond strength.

Aspect Ratio & Fiber Volume Fraction (%) & Mix Type	Theoretical Bond Strength, N/mm^2^			
	IS 456: 2000		BS 8110	
	Plain Bar	Deformed Bar	Plain Bar	Deformed Bar
0 (0%) CC	1.40	2.24	1.53	2.74
83.33 (3%) CC			1.85	3.30
0 (0%) CSC			1.41	2.52
66.67 (3%) CSC			1.53	2.74
